# Social Robotics in Therapy of Apraxia of Speech

**DOI:** 10.1155/2018/7075290

**Published:** 2018-03-11

**Authors:** José Carlos Castillo, Diego Álvarez-Fernández, Fernando Alonso-Martín, Sara Marques-Villarroya, Miguel A. Salichs

**Affiliations:** Departamento de Sistemas y Automática, Universidad Carlos III de Madrid, 28911 Madrid, Spain

## Abstract

Apraxia of speech is a motor speech disorder in which messages from the brain to the mouth are disrupted, resulting in an inability for moving lips or tongue to the right place to pronounce sounds correctly. Current therapies for this condition involve a therapist that in one-on-one sessions conducts the exercises. Our aim is to work in the line of robotic therapies in which a robot is able to perform partially or autonomously a therapy session, endowing a social robot with the ability of assisting therapists in apraxia of speech rehabilitation exercises. Therefore, we integrate computer vision and machine learning techniques to detect the mouth pose of the user and, on top of that, our social robot performs autonomously the different steps of the therapy using multimodal interaction.

## 1. Introduction

Apraxia of speech (AOS) is a neurological disorder in which the messages from the brain to the mouth are disrupted, and the person cannot move his/her lips or tongue to say sounds properly. This condition is caused by a damage in the left hemisphere of the brain generated by strokes, Alzheimer's or brain traumas, among others. The severity of the apraxia depends on the nature of the brain damage. AOS is also known as acquired apraxia of speech, verbal apraxia, and dyspraxia [1].

The focus of intervention is on improving the planning, sequencing, and coordination of muscle movements for speech production. The muscles of speech often need to be “retrained” to produce sounds correctly and sequence sounds into words. Exercises are designed to allow the person to repeat sounds over and over and to practice correct mouth movements for sounds [2]. Currently, there are three different interventions for AOS rehabilitation: (i) intervention based on motor control: these exercises consist of producing phonemes and sequences of phonemes through accurate, controlled, and conscious movements. The aim of these therapies is to automate such movements to be subsequently performed unwittingly; (ii) intervention based on augmented systems: these methods include several input channels to improve the therapy results. Audio and images are traditionally mixed to help remembering how to pronounce difficult and long words; and (iii) interventions based on melodies: these therapies are adopted in patients that preserve an auditive comprehension of the language. In these cases, the patient has to imitate different melodies proposed that remark the stressed syllables of the wards, establishing the rhythm of the melodies [3]. These interventions are frequently planned as intensive, one-on-one speech-language therapy sessions for both children and adults. Thus, the repetitive exercises and personal attention needed to improve AOS are difficult to deliver in group therapy [4].

In recent years, robots are gaining popularity in rehabilitation therapies, mainly in traumatology, where the robot holds the user's weight or helps moving a determined limb. Robots have been proven to be effective in assisting the therapist to provide safe and intensive rehabilitation training for the stroke subjects. Nevertheless, in the general setting of these systems, a therapist is still responsible for the nonphysical interaction and observation of the patient by maintaining a supervisory role of the training, while the robot carries out the actual physical interaction with the patient. In most applications, rehabilitation robots have been mainly employed in lower and upper limb therapy [5–8].

Rehabilitation using robotics is generally well tolerated by patients and has been found to be an effective adjunct to therapy in individuals suffering from motor impairments, especially due to stroke. Therefore, we believe that robotics can be introduced to other rehabilitation areas such as AOS. To the extent of our knowledge, this proposal is innovative as robotic technologies have not been applied to this field so far. We propose following the first kind of intervention presented in this section in which the user repeats exercises to practice mouth movements; in our case, we take inspiration from mouth poses associated to the five vowels in the Spanish tongue. Here, “a” is pronounced like the “a” in the word “father” (/a/); “e” is pronounced like the “a” in the word “date” (/e/), except that it is shorter and crisper; “i” is pronounced like the “ee” in the word “see” (/i/); “o” is pronounced like the “o” in the word “no” (/o/); and “u” is pronounced like the “e” in the word “new” (/u/). Thus, we propose using some of these sounds because their pronunciation imply different poses of the mouth, associated to a range of muscular movements.

We believe that a social robot could help in AOS therapy offering a new and eye-catching way of assisting in the exercises. The robot adds to the therapy some new resources such as a screen to stimulate the patient, offering a visual reinforcement to the exercises. Additionally, the human-robot interaction (HRI) capabilities of a social robot could enhance the traditional therapy, maximizing the human resources while keeping a personalized treatment. That is, a therapist could take care of more patients having robots develop parts of the treatments.

We propose using machine learning techniques for vowel pose recognition and identification. The input information is collected by an RGB-D device, a *Microsoft Kinect*, and with this information, the system obtains mouth poses which are used in the exercises to guide the users. Interaction is performed through a multimodal system that integrates body expressions, voice interaction, and a graphical user interface (GUI), and all of these modalities are developed to give instructions to the patient as well as encourage him/her during the exercise.

The rest of this manuscript is structured as follows: Section 2 provides the insights of current therapies for AOS, presents new robotic developments for physical therapy and cognitive rehabilitation, and analyses some face detection and classification techniques related to our approach. Next, Section 3 presents the details of our proposal, describing its main phases.  Section 4 presents the experiments conducted to validate our work along with the robotic platform, the social robot Mini, and the metrics for evaluating the approach. This section also presents the preliminary results from integrating and testing the AOS exercises in the social robot. Finally, Section 5 analyses the main contributions or our work and draws the main conclusions.

## 2. Related Work

The ability of speech is commonly affected after suffering Alzheimer's, dementia, or a stroke. Traditional speech therapies focus on mitigating this problem in case of cognitive impairment or rehabilitating in case of cerebrovascular accidents. The recovery time in these cases is around three years [9] in which speech therapy yields positive results in most cases. Apart from this line of therapy, there are others such as music therapy that are usually applied to patients with neurological problems, generally elders. In the case of music, the therapy consists of patients emitting singing and emitting sounds from given melodies in order to improve pitch, variability, and intelligibility of speech [10]. Tomaino and Sacks [11] demonstrated that music therapy helps reorganizing the brain function in patients with brain alterations.

Apart from traditional therapies, technology is being incorporated to health environments. More precisely, robotics is gaining importance, mainly in the fields of physical therapy and cognitive rehabilitation. In these cases, exercises are supervised by therapists who are in charge of selecting the tasks to perform and monitor the procedure [12]. The robot Paro is a good example of the application of robots in cognitive therapy. It imitates a baby harp seal and has been used in therapy with elder people with dementia, increasing the willingness of patients to communicate and a steady increase in physical interaction, not only between patients and the robot but also among patients as well [13, 14]. Another robotic platform used in cognitive therapy is Babyloid, a baby-like robot designed to be taken care of [15]. This robot is intended for recreational therapy in which the robot becomes a pet instead of an animal. These proposals are mainly intended for interaction with elderly people with moderate cognitive impairments.

Other robotic platforms provide a higher degree of interaction in therapies with people with mild cognitive problems. This is the case of Eldertoy [16], a robot developed to achieve both entertaining and gerontological capabilities. This robot offers different interaction channels to communicate with users: gestures, voice, touch-screen, and external actuators. Therapy with this robot is conceived through manipulation and display multimedia content. Therefore, therapy specialists are furnished with a tool able to run games by using the sensors integrated in the platform. The robot Mini is another proposal for therapy with elders in early stages of Alzheimer's or dementia [17, 18]. Mini is a plush-like desktop robot that offers functionalities related to safety, personal assistance, entertainment, and stimulation. In this work, we aim to extend the capabilities of Mini to conduct speech therapy. More details about the robot design and features can be found in Section 3.

Another research area integrated in our work is computer vision. The literature offers several approaches for face detection and recognition [19, 20]. Applications range from people recognition [21], surveillance [22] to emotion detection and regulation [23, 24]. Although there are several techniques to retrieve facial features, this problem is still challenging since most of the approaches are highly dependent on the face orientation. In this work, we have integrated Stasm, an active shape model-based approach coupled to a support vector machine (SVM) classifier that retrieves facial features [20]. Out of these features, the user's mouth is represented with 18 3D points, which will be the input for the machine learning algorithm.

Apart from detecting the mouth, recognizing the mouth pose is crucial to have an algorithm that can be integrated into a speech therapy application. Machine learning has been widely applied in face recognition and recognition of facial expressions [25, 26]. Within the number of techniques, SVM, Adaboost, linear discriminant analysis or, more recently, deep learning [27], among others, try to cope with known problems such as different poses, illumination, ages, and occlusions that nowadays still pose a challenge. In our work, we test several classifiers integrated within Sci-Kit learn [28], an open-source machine learning library written in Python language. It provides feature classification, regression, and clustering algorithms.

## 3. Materials and Methods

**Figure 1:**
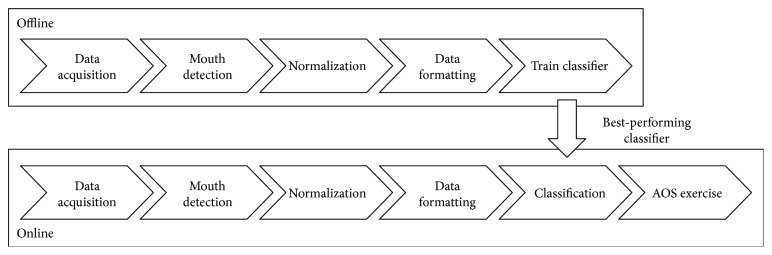
Proposed approach pipeline. The upper path corresponds to the offline analysis for assessing the best classifier. The lower path corresponds to the software running in the robot with the speech therapy exercise.

This section presents the details of the proposed approach that allows a social robot, equipped with a 3D camera, to conduct an AOS exercise autonomously.  Figure 1 shows the main steps of our proposal, which is roughly divided into two operation modes. First, we need to assess the classifier that performs best for our kind of data. In this process, we acquire information from users, preprocess it, and train a set of classifiers to select the best-performing one. This classifier is used next online, thus integrated in the robotic platform where the speech therapy application uses the mouth pose detected to conduct the exercises. Note that the four first steps are the same in both approaches.

### 3.1. Mouth Detection from RGB-D Data

**Figure 2:**
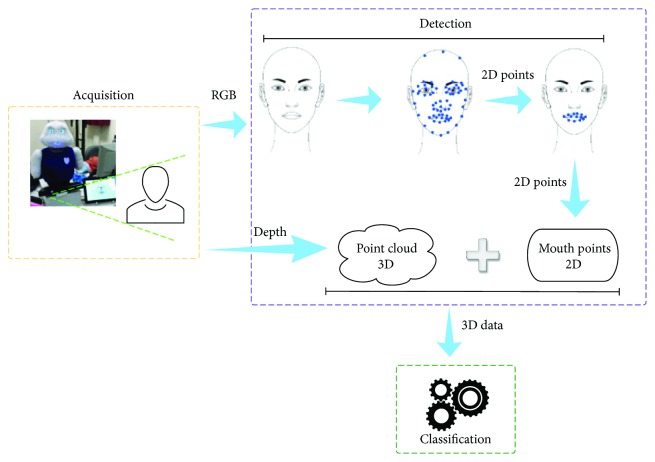
Mouth detection pipeline using Stasm.

The system described here uses a Microsoft Kinect, which provides *RGB* images and depth data synchronized both in terms of time and field of view. After information acquisition is performed, the system extracts face features in *2D* using the open-source library *Stasm* [20]. Then, those features are translated into *3D* points which are finally classified to recognize the mouth pose (see Figure 2).

The mouth detection process is composed by two main steps. Data acquisition is performed through a middleware specifically designed to work with *RGB-D* devices, *OpenNI* (OpenNI website: http://openni.ru/). Two information flows are generated from the Kinect device: an *RGB* image stream and a point cloud containing depth information. Next, the system processes the *RGB* data to identify the mouth within a detected face using *Stasm*. This library characterizes a face with 77 points of which 18 belong to the mouth. These points are next matched to the depth information from the camera and formatted to be used in the next phase, mouth pose classification. A more detailed description of the mouth detection system can be found in a previous work [29].

### 3.2. Machine Learning Tools for Mouth Pose Classification

In our approach for mouth pose recognition, we aim to test a series of classification techniques integrated within Scikit-Learn [28]. For the classifier selection, we take as a starting point a previous work [29] in which mouth detection was evaluated using WEKA [30], a well-known data mining tool that allows preprocessing, classification, regression, clustering, association rules, and visualization of data. In this case, we wanted to take the study one step further and integrate the best-performing classifier in our robotic platform (WEKA was not directly integrated within the framework ROS). Thus, we compared the performance of the following classifiers:

- *k-Nearest neighbours* (*k*-NN) is a nonparametric method used for classification and regression in which the input consists of the *k* closest training examples in a feature space [31]. In our problem, the output is a mouth pose where a sample is classified by a majority vote of its neighbours with the object being assigned to the most common class among its k-nearest neighbours.
- *Support vector machine* is a supervised learning technique for classification and regression that builds a hyperplane or set of hyperplanes in a high- or infinite-dimensional space [32]. An SVM can perform linear and nonlinear classification mapping the inputs into high-dimensional feature spaces.
- *C4.5* is an algorithm that generates a decision tree from a set of training data using the concept of information entropy [33]. At each node of the tree, the algorithm chooses the attribute of the data that most effectively splits the set of samples into subsets enriched in one of the classes. The splitting criterion is the normalized information gain (difference in entropy).
- *Random forest* is an ensemble learning method for classification and regression that construct multiple decision trees at training time and outputs the class that corresponds to the mode of the possible classes (mouth poses). An advantage of random forest is that this technique mitigates the overfitting problem caused by traditional decision trees [34].

### 3.3. Assessing the Best Classifier: Offline Analysis

Before addressing the logic of the speech therapy exercise, it was necessary establishing which classifier offered better performance with our input data. This operation mode, depicted in the upper path of Figure 1, starts with a data acquisition phase in which the RGB-D device provides colour images and point clouds with the 3D representation of the scene. The next step, mouth detection, works as described in Section 3.1, using Stasm to generate a 3D array of 18 points corresponding to the mouth detected in the input data.

Since the head position in the image varies as the user moves, it is important to normalize the data to establish a common frame for reference. Thus, the normalization step computes the centroid of the mouth, setting it as the origin of coordinates for the 18 points (see (1), (2), and (3)). Each one of these points is defined by its <*x*, *y*, *z*> components, and therefore, (4), (5), and (6) show that the normalization for each of them is calculated with respect to the centroid.

(1) $$\begin{array}{c} x_{centroid}=\frac{1}{18}\overset{18}{\underset{1}{\sum }}x_{i}, \end{array}$$

(2) $$\begin{array}{c} y_{centroid}=\frac{1}{18}\overset{18}{\underset{1}{\sum }}y_{i}, \end{array}$$

(3) $$\begin{array}{c} z_{centroid}=\frac{1}{18}\overset{18}{\underset{1}{\sum }}z_{i}, \end{array}$$

(4) $$\begin{array}{c} x_{i_{normalized}}=x_{i}-x_{centroid}\;\forall i\in \left[1,18\right], \end{array}$$

(5) $$\begin{array}{c} y_{i_{normalized}}=y_{i}-y_{centroid}\;\forall i\in \left[1,18\right], \end{array}$$

(6) $$\begin{array}{c} z_{i_{normalized}}=z_{i}-z_{centroid}\;\forall i\in \left[1,18\right]. \end{array}$$

These normalized points are formatted in tuples for the classifier. Each tuple composed by 54 values plus the class for each pose was recorded. After the data is formatted, we trained the classifiers previously described in Section 3.2.

### 3.4. Online Execution

The best-performing classifier identified in the previous section is integrated in the online execution mode of our system, described in the lower path of Figure 1. The four first steps are common to both offline and online executions as they are intended for data acquisition, detecting the points corresponding to the mouth and normalize them as well as formatting the data for classification. In the online mode, the data formatted is then processed in a classifier which output is used in the AOS exercise to assess the user performance and guide him/her during the session.

**Figure 3:**
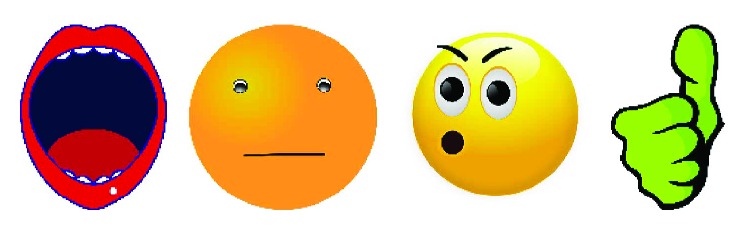
Images to give feedback about the mouth pose and the user's performance. First, image to indicate how to make the “a” pose. Second, image to indicate how the closed-mouth pose should be. Third, image to indicate how to make the “u” pose. Fourth, image to congratulate the user.

When one of the three poses reaches a number of detections, the system selects that pose as the current one and interacts with the user, expressing congratulations in case the pose detected was the one expected, or correcting the user if another pose is detected. A repertoire or corpus of utterances has been created to congratulate and correct the user (see Table 1) as a complement to the images shown in the tablet (see Figure 3). Additionally, the robot expresses gestures with its body to help engaging the user in the exercise. When the user fails to perform the pose, a sad expression is performed while otherwise the robot shows a happy expression (Instead of denning here what they are about, a demo video has been released in which those gestures are clearly demonstrated. The video link is presented at the end of Section 4.4).

In the current version of the system, the process of detecting a mouth pose and congratulating/correcting the user is repeated three times although the system is flexible enough to change and adapt the exercise logic.

## 4. Results and Discussion

### 4.1. Robotic Platform: Mini

**Figure 4:**
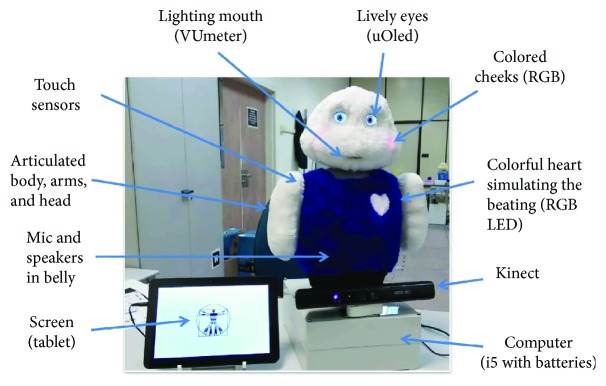
Mini, the social robot involved in the experiments [40]. Apart from its plushy shape, the robot is equipped with a series of sensors and actuators for HRI.

The system developed in this work was integrated in Mini, a desktop social robot designed and built at RoboticsLab research group from the Charles III University of Madrid (see Figure 4). Originally, this robot was designed to interact with elder people with mild cognitive impairment [18]. Nevertheless, the capabilities of this platform allows other users and applications such as our current goal.

Mini is equipped with multiple HRI interfaces including automatic speech recognition (ASR) [35], voice activity detection [36], emotion detection [37], a text to speech (TTS) system, a tablet, and an RGB-D device. Moreover, Mini possesses 5 degrees of freedom to allow moving its arms, base, and head. The interaction capabilities complete with touch sensors, two uOled screens for the eyes, RGB LEDs in the cheeks and heart, and a VUmeter as mouth to create the illusion of a talking robot. All of these interfaces are integrated in a natural dialogue management system [38] which enables the robot to carry out natural interactions. Finally, these components are integrated using ROS framework [39].

### 4.2. Metrics for Evaluating the Classifiers

Since our classifiers have to solve a multiclass problem, the metric selected for assessing the best one was the macroaverage *F*-score. Macroaverage means that the metric is independently computed from each class and then the average is calculated. This metric uses the precision and recall for each class and calculates the mean precision and recall of all classes as shown as follows:

(7) $$\begin{array}{c} \bar{Precision}=\frac{\sum_{i=1}^{N}{TP}_{i}}{\sum_{i=1}^{N}{TP}_{i}+{FP}_{i}},\\ \bar{Recall}=\frac{\sum_{i=1}^{N}{TP}_{i}}{\sum_{i=1}^{N}{TP}_{i}+{FN}_{i}}, \end{array}$$

where *N* is the total number of classes, TP_i_ corresponds to the true positives achieved in class i, FP_i_ are the false positives for class i, and FN_i_ are the false negatives in class i. Then, the macroaverage *F*-score is computed as the harmonic mean of these two values as shown as follows:

(8) $$\begin{array}{c} Macroaverage\_F‐score=\frac{2\times \bar{precision}\times \bar{recall}}{\bar{precision}+\bar{recall}}. \end{array}$$

### 4.3. Experiments

**Figure 5:**
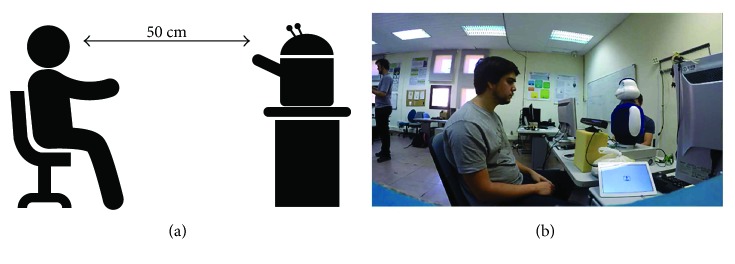
Experimental setup. The user was sitting in front of the robot at 0.5 meters, a natural distance for interaction that ensures clear images of the face.

In our experiments, users sit in front of the robot, at 0.5 meters (see Figure 5(a)). A previous study indicated that at a range of 0.5 meters, the detector performance in order to locate the mouth accurately in the face was high, suffering a degradation with the distance that at 2 meters was too poor to achieve reliable detections [29]. Moreover, as shown in Figure 5, right, this distance allows a natural interaction with the robot, for instance with its tablet that is usually placed between the robot and the user. Note that in Figure 5(b), the tablet is on the left side of the robot. In this specific case, the tablet was placed there just for illustrative purposes. Also, the Kinect camera changed the usual location (see Figure 4) to allow a better acquisition of face images.

The following sections detail the experiments conducted to assess the performance of our system and its feasibility for speech therapy. Also, the aim of this set of tests was to select the best classifier for our data. We first tested the performance with the most different poses “a” and “u”, as described in experiment 1. For our second experiment, we added a new “neutral” pose that corresponded to the mouth closed and carried out experiment 2. 14 users were involved in our experiments, and the method for dividing the datasets was 1-fold cross-validation with 60% of the instances for training and 40% for test.

#### 4.3.1. Experiment 1: Training 2 Poses

**Figure 6:**
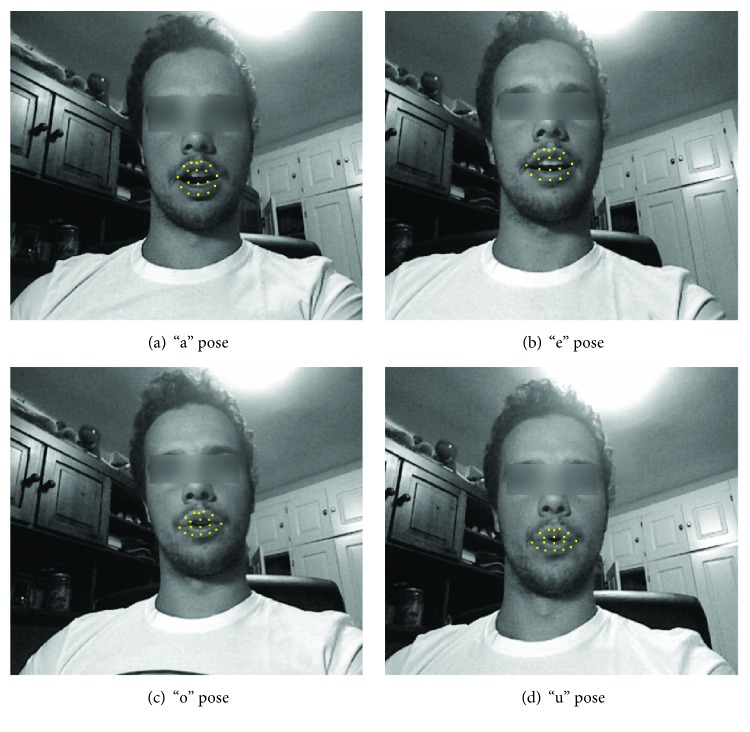
Mouth points as detected by Stasm. We can see in this example how there is low variability between some mouth poses.

This experiment is meant to assess the feasibility of the classifiers described in Section 3.2 to distinguish between two mouth poses. Although this set of poses may seem reduced, they are different enough as to implement a range of mouth movements that could be useful in SOA therapy. Moreover, recognizing the mouth is not an easy task, leading to similar representations of different poses as shown in Figure 6. The two first images correspond to the poses associated to “a” and “e,” and the two last ones correspond to “o” and “u.”

In this test, the dataset was composed of 1200 instances for the pose “a” and 1425 for the “u” pose (see Table 2). After the test, two classifiers, C4.5 and SVM, showed promising results, with a macroaverage *F*-score of 0.82 and 0.81, respectively, as shown in Table 3. Additionally, Table 4 offers the confusion matrix for the best classifier in this experiment, C4.5, in which we can observe an accuracy that starts to be competitive for our speech therapy application.

#### 4.3.2. Experiment 2: Training 3 Poses

In this experiment, a new pose was added to the dataset, mouth closed, to complement the cases for the AOS exercise. Therefore, a new class, mouth closed, was added to our dataset with 3624 instances. For the previous classes, new instances were added as well, having a total of 4623 instances for the “a” pose and 5250 instances for the “u” pose. Finally, the classifiers were retrained with the new data. The results show that C4.5 is again the best classifier, with k-NN offering competitive performance (see Table 3). Therefore, since C4.5 showed the best behaviour in both experiments, this classifier is the one selected for the online execution.

In this experiment, the results improved with respect to the previous one as shown in the confusion matrix for C4.5 classifier (see Table 5). Here, the recognition rate for the “a” pose reached 95%; in case of the “u” pose, the rate is 93%, and finally for mouth-closed pose, the rate is 99.67%.

### 4.4. Integration in the Social Robot

This section analyses the performance of the detection and classification integrated with the speech therapy application. In this case, users trained the system online in periods ranging between 5 and 10 minutes for the three poses together.  Table 6 shows the performance of our pretrained C4.5 classifier when offered new data. We can see how in some cases as in mouth-closed pose that the performance drops to the point that the system is not usable.

At this point, we realised that we needed to perform some training with the new users' data, but in this case, that training should not be as intensive as in previous experiments. Since the final application is speech therapy, we cannot expect that users will be willing to train the robot for long periods of time. In this case, the users are trained from the system with online detections in periods ranging between 5 and 10 minutes for the three poses together. We believe that this would not cause boredom or fatigue in the users as it only needs to be performed once per new user. With this new data, the performance of the system improves to levels comparable to experiment 1 (see Table 7), and although not reaching the scores achieved in experiment 2 with cross-validation, these levels are high enough to ensure a good detection rate.

In the online execution, we experimentally set the score threshold to consider valid detection to 0.35 and a pose was output after six successful recognitions. In most cases, we noticed that the detection score was close to one, dropping to low values for missdetections. The number of successful recognitions to consider a valid pose directly impacts the execution time of our system since a bigger number would cause a slow response and a smaller number could lead to wrong detections. Therefore, six valid detections were considered as a good tradeoff between time of response and accuracy.

**Figure 7:**
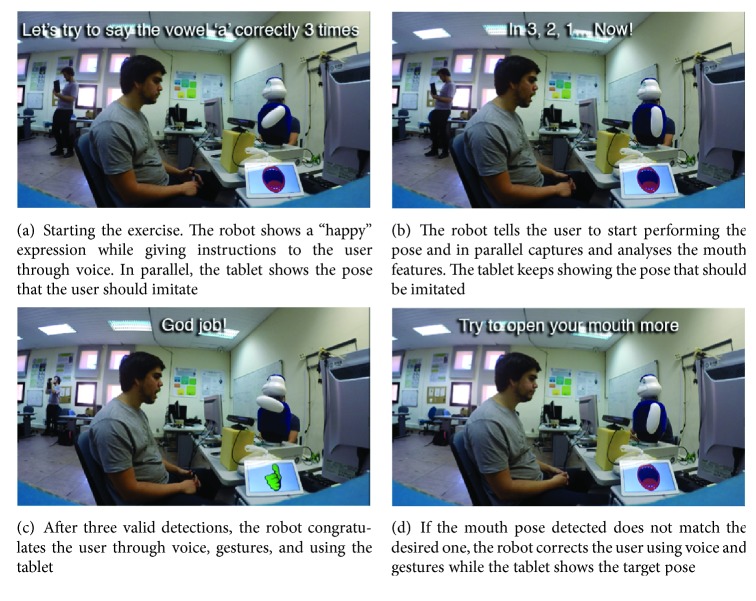
Running example of the speech therapy proposal. The robot leads the user through the exercise, encouraging him to keep participating.

Figure 7 offers an overview of the speech therapy proposal with the different phases where the robot guides the user along the exercise. First, the robot provides a simple explanation about the exercise (see Figure 6) using gestures, voice, and the tablet to convey the messages. Next, the exercise starts and the user should start making the desired pose while the system is detecting and classifying the mouth pose (see Figure 6). Finally, after three successful detections, the robot congratulates the user (see Figure 6). There is also the possibility that the system does not detect the target pose. In this case, the robot corrects the user, explaining how to achieve the desired pose (see Figure 6). Along this exercise, the robot uses voice, gestures, and the tablet to give instructions and feedback to the user. A video has been uploaded with more details about the execution of the system which can be found in https://youtu.be/XRrIP3BcwCY.

### 4.5. Discussion

The results and the experimental conditions of the proposed approach are summarized in Table 8. These results show how our proposal provides high accuracy for mouth pose classification, up to 0.95 in the cross-validation test. It is worth remarking that the experimental phase in this paper is intended as a proof of concept and that we are currently working on testing it with real users. Additionally, we are aware that mouth poses could change when working with people with motor mouth problems and that this fact could affect the performance of the classifiers. In this regard, our plan is to add real user data and retrain the system when deploying it in real scenarios.

Also, the set of mouth poses recognized may seem too small but for AOS therapy purposes, their differences were considered enough for a first approach. It is our intention involving experts to evaluate the feasibility of our proposal both in terms of poses recognized and the dynamic of the exercise.

## 5. Conclusions

This manuscript introduced an approach for apraxia of speech therapy using a social robot. The system consists of two main phases: an *offline* one in which we train a set of classifiers after detecting and normalizing the mouth information from users and an *online* one that runs in our social robot Mini. This operation runs in real time, integrating the best-performing classifier, and guides the user through an AOS exercise.

The experiments included up to three mouth poses (“a”, “u,” and mouth closed) which we consider are enough for a first approach of therapy for their differences regarding the mouth positions. The classifiers trained on our dataset are composed by information from 14 users in our experiments offline, to assess the best one. In these offline experiments, C4.5 was the best classifier for our data (achieving 0.95 of macroaverage *F*-score), and therefore, it was integrated within the final approach. In the online tests with the whole system integrated in the social robot, we conducted additional experiments with 7 new users, the first one running the system with untrained data which showed a performance decrease in the mouth poses classification. This motivated the second experiment in which we retrained the classifier adding a small set of samples from the new users. In this case, the performance rose again to competitive values.

We believe that the results achieved in our experiments are promising, and thus, we are intended to proceed with the next stage: testing the AOS exercise with real users and therapists.

To the extent of our knowledge, this proposal is innovative as robotic technologies have not been applied to this field so far.

## Figures and Tables

**Table 1:** Set of utterances to convey messages during the exercise (approximate translation from Spanish).

Situation	Sentence	Details
Congratulating	Very good	General congratulation utterances
	Keep going	
	You are doing great	
Correcting	Open a little less your mouth	Corrections in case the user is opening the mouth more than expected
	Open your mouth less	
	Please, close your mouth a bit	
	Open your mouth a bit more	Corrections in case the user is opening the mouth less than expected
	Open your mouth a bit more	
	Make a bigger mouth	
	You are almost there	Utterance to encourage the user during the exercise
	Keep trying	
Starting exercise	Let us try to say “a” correctly three times	Practicing with “a” pose
	Let us try to say “u” correctly three times	Practicing with “u” pose
	Try to keep your mouth closed for three rounds	Practicing with mouth closed pose
	In 3, 2, 1... Now!	Start signal

**Table 2:** Dataset summary for experiments 1 and 2.

Pose	Experiment 1 instances	Experiment 2 instances
“a”	1200	4623
“u”	1425	5250
Mouth closed	N/A	3624
Total	1625	13497

**Table 3:** Results for experiments 1 and 2. Macroaverage *F*-score for the four classifiers tested with two mouth poses.

Classifier	Experiment 1: macroaverage *F*-score	Experiment 2: macroaverage *F*-score
Random forest	0.57	0.47
C4.5	**0.82**	**0.95**
k-NN	0.54	0.93
SVM	0.81	0.63

**Table 4:** Experiment 1: confusion matrix for C4.5 classifier identifying 2 poses.

	Predicted A	Predicted U
Actual A	86%	14%
Actual U	12%	88%

**Table 5:** Experiment 2: confusion matrix for C4.5 classifier identifying 3 poses.

	Predicted A	Predicted U	Predicted mouth closed
Actual A	95%	4%	1%
Actual U	5%	93%	2%
Actual mouth closed	0.03%	0.3%	99.67%

**Table 6:** Confusion matrix for the first test with untrained users.

	Predicted A	Predicted U	Predicted mouth closed
Actual A	52%	32%	16%
Actual U	36%	40%	24%
Actual mouth closed	0%	90%	10%

**Table 7:** Confusion matrix for the test retraining the classifier with new user data.

	Predicted A	Predicted U	Predicted mouth closed
Actual A	85%	12%	3%
Actual U	8%	75%	17%
Actual mouth closed	0%	14%	86%

**Table 8:** Numbers summarizing the experimental conditions and results of our proposal.

Recognized poses	**3 poses** (“a,” “u,” mouth closed)
Dataset instances	**1625** for experiment 1 and **13497** for experiment 2
Input features per instance	**54 features**: 18 mouth points ^∗^ 3 components (x, y, z)
Users involved in the dataset	**14 users** for the 2 datasets
Classifiers tested	**4 classifiers**
Metric for comparison	**Macroaverage *F*-score**
Best algorithm for classifying poses	**C4.5** (0.81 and 0.95 of macroaverage *F*-score in the experiments)
Users involved in the real tests	**7 users**
